# Experience in Diagnostic of HIV Drug Resistance in the Mekong Delta Region, Vietnam: A Comparative Analysis Before and After the COVID-19 Pandemic

**DOI:** 10.3390/diagnostics15101279

**Published:** 2025-05-18

**Authors:** Huynh Hoang Khanh Thu, Alexandr N. Schemelev, Yulia V. Ostankova, Vladimir S. Davydenko, Diana E. Reingardt, Ton Tran, Le Chi Thanh, Thi Xuan Lien Truong, Areg A. Totolian

**Affiliations:** 1 Pasteur Institute in Ho Chi Minh City, Ho Chi Minh City 70000, Vietnam; thuhhk@pasteurhcm.edu.vn (H.H.K.T.); tont@pasteurhcm.edu.vn (T.T.); thanhlc@pasteurhcm.edu.vn (L.C.T.); 2 Saint Petersburg Pasteur Institute, St. Petersburg 197101, Russia; shenna1@yandex.ru (Y.V.O.); vladimir_david@mail.ru (V.S.D.); dianavalutite008@gmail.com (D.E.R.); totolian@spbraaci.ru (A.A.T.); 3 Faculty of Pharmacy, Van Lang University, Ho Chi Minh City 70000, Vietnam; lien.ttx@vlu.edu.vn

**Keywords:** HIV-1, virological failure, HIV drug resistance mutation, NRTI, NNRTI, PI, Vietnam

## Abstract

**Background:** Vietnam has made significant strides in reducing the prevalence of HIV infection and achievements in its antiretroviral treatment program. However, the COVID-19 pandemic and financial challenges in the healthcare system have posed significant obstacles to maintaining effective HIV treatment and monitoring, particularly among vulnerable populations. This study aims to evaluate the situation of HIV drug resistance among patients who have experienced treatment failure in the Mekong Delta region and to compare data from 2019 to 2022. **Methods:** The study material was blood plasma samples from HIV-infected individuals with ART failure: 316 collected in 2019 and 326 collected in 2022. HIV-1 genotyping and mutation detection were performed based on an analysis of the nucleotide sequences of the *Pol* gene region. A total of 116 HIV-infected individuals with virological failure in 2019 and 2022 were assessed for HIV drug resistance. **Results:** The study revealed a high proportion of participants with viral loads exceeding 1000 copies/mL, significantly increasing from 12.0% in 2019 to 23.9% in 2022 (OR = 2.3; *p* = 0.0001). HIV drug resistance mutations were detected in 84.21% of cases in 2019 and 92.59% in 2022. The prevalence of concurrent resistance to NRTIs and NNRTIs was 37.5% and 30.13% in 2019 and 2022, respectively. There was a statistically significant decrease in NNRTI resistance (OR = 0.32, χ^2^ = 5.43, *p* < 0.05). In contrast, multi-drug resistance to protease inhibitors rose from 18.52% to 45.21% (φ* = 0.00403, *p* < 0.05). Triple-class resistance was identified only in 2022 (17.81%). The most common mutations included M184I/V, D67N, K103N, Y181C, and V82A/S/T, with D67N rising significantly from 3.13% to 21.92%. The predominant subtype was CRF01_AE. **Conclusion:** A high prevalence of viral non-suppression and HIV drug resistance was observed among patients in the Mekong Delta region, particularly after the onset of the COVID-19 pandemic. Our study highlights the ongoing challenges that the HIV/AIDS treatment program in Vietnam must address in the post-pandemic period to sustain its success and achieve the goals of the country’s HIV prevention strategies.

## 1. Introduction

Human immunodeficiency virus (HIV) causes a slowly progressive disease leading to acquired immunodeficiency syndrome (AIDS). The spread of HIV has by now become a global problem and a de facto pandemic. According to the latest figures released by UNAIDS, the global number of people living with HIV (PLHIV) in 2023 is 39.9 million. The number of new HIV infections is 1.3 million, about 39% lower than in 2010 but well above the 2025 target [1].

The introduction of highly active antiretroviral therapy (ART) has significantly improved the prognosis for HIV-infected patients by suppressing viral replication, reducing mortality, and increasing survival. This treatment has effectively turned an inevitably fatal disease into a manageable chronic condition by lowering viral loads to undetectable levels and increasing CD4+ T-lymphocyte counts [2]. The rising death toll among HIV-infected individuals is primarily due to inadequate access to ongoing care and treatment. However, with consistent treatment, the prognosis is generally favorable, significantly reducing the risk of disease progression and limiting the spread of the virus.

Despite improved HIV/AIDS rates with ART, viral drug resistance remains a significant threat due to the virus’s high mutation rate, genetic variability, recombination of the reverse transcriptase enzyme, and the uneven rate of fixation of nucleotide substitutions during molecular evolution on ART, which lead to the accumulation of drug resistance mutations over time [3,4].

Furthermore, such factors as low adherence to therapy, lack of control of viral load level in peripheral blood, and unjustified changes in the therapy regimen increase the risk of viral drug resistance [5]. Acquired HIV drug resistance (ADR), which results from mutations during viral replication in individuals on antiretroviral therapy, is one of three primary categories of HIV drug resistance (HIVDR) recommended by the World Health Organization (WHO) for national surveillance [6]. In fact, high levels of acquired HIV drug resistance, exceeding 80%, were observed for both non-nucleoside and nucleoside reverse transcriptase inhibitors (NNRTIs and NRTIs) between 2014 and 2020 worldwide [7]. HIV resistance to several classes of drugs is of particular importance, as it significantly reduces treatment options.

Vietnam has achieved remarkable success in bringing down the prevalence of HIV infection in the population to under 0.3%. Over the past decade, the country has witnessed a significant reduction in the number of new infections and HIV/AIDS-related deaths, decreasing by more than two-thirds compared to a decade ago [8,9]. At the end of 2023, there were 230.344 PLHIV [10]. Vietnam’s HIV program targets the UNAIDS 95-95-95 goals, but progress has been uneven: 88% of PLHIV were aware of their status, 80% were on ART, and 98.3% of those on ART achieved viral suppression by the end of 2023 [11].

ART was first introduced in Vietnam in the mid-1990s and has been rapidly scaled up since 2005, with a total of 178,928 people receiving ART by the end of 2023 [11]. Over time, Vietnam’s national guidelines for ART regimens have seen significant updates. Prior to November 2019, the recommended first-line treatment regimen for adults consisted of a combination of two NRTIs and one NNRTI, with the preferred regimen being TDF/3TC/EFV [12,13]. Since the end of 2019, the preferred first-line ART regimen has transitioned to a fixed-dose combination of two NRTIs and one integrase strand transfer inhibitor (INSTI) for patients initiating ART [14,15]. This shift reflects advancements in treatment strategies aimed at improving patient outcomes and reducing the likelihood of drug resistance to NNRTIs. For patients experiencing treatment failure, the second-line ART regimen substitutes the NNRTI or INSTI with a protease inhibitor (PI), typically LPV/r [12,13,14,15]. Monitoring ART effectiveness is critically dependent on viral load testing. Patients on ART undergo viral load testing every six months during the first year of treatment, and then annually thereafter. Virological failure is defined as a confirmed viral load of more than 1000 copies/mL after at least six months on ART, despite good adherence to therapy.

Since the widespread implementation of ART, Vietnam has published national surveillance reports on HIVDR three times, covering the periods of 2014, 2017–2018, and 2019–2020. The percentage of viral load suppression reported in these surveys ranged from 94.6% to 96.1%. ADR prevalence consistently remained below 5% across all these surveys. In 2013, 4.6% of individuals on ART for at least 36 months experienced ADR [12]. By 2017 and 2019, this rate decreased to 2.5% and 3.0% for people who had been on ART for 12 months, and 2.7% and 3.4% for those on ART for at least 48 months, respectively. The most common drug resistance mutations (DRMs) identified in these surveys included Y181C, K103N, and G190A, which confer resistance to NNRTIs as well as M184V, V75M, and several thymidine analog mutations (TAMs), such as T215F/I/Y, K219E/Q, K70R, D67N, M41L, and L210W [7,16,17,18]. To date, no surveillance data on HIVDR related to integrase strand transfer inhibitors (INSTIs) have been reported.

In recent years, the Vietnamese Ministry of Health has been facing challenges in transitioning the financial responsibility for HIV services from international donors to the domestic governmental budget by integrating these services into the social health insurance (SHI) system, which has posed some challenges. Vulnerable populations, including those in remote areas like the Mekong Delta and Northern Highlands, face challenges in maintaining consistent treatment and monitoring. These factors may contribute to non-adherence, viral non-suppression, and an increased risk of developing HIVDR [19,20]. Moreover, the onset of the COVID-19 pandemic from 2020 to 2022 placed a severe strain on Vietnam’s health systems. Measures such as social distancing, travel restrictions, and lockdowns aimed at controlling the spread of the virus likely disrupted health service delivery, including HIV care and treatment programs. As of now, there has been no national surveillance or study on HIVDR among patients on ART since the pandemic began.

In light of these circumstances, our study aims to assess the HIV drug resistance situation among patients who have experienced virological failure in the Mekong Delta region and compare this situation between 2019 and 2022.

## 2. Materials and Methods

The study protocol received approval from the Ethics Committees at both the Saint Petersburg Pasteur Institute and the Pasteur Institute in Ho Chi Minh City. The study material was 316 blood plasma samples collected in 2019 (age ranged from 18 to 65 years; male and female percentages were 57.91% and 42.08%, respectively) and 326 samples collected in 2022 (age ranged from 20 to 63 years; male and female percentages were 66.87% and 33.12%, respectively). The samples were collected from treatment facilities in southern Vietnam and deposited at the Pasteur Institute in Ho Chi Minh City. All samples were obtained from HIV-infected individuals on antiretroviral therapy with suspicious clinical manifestations and/or those with insufficient adherence. All samples were transferred anonymously.

All studies were performed as previously described [21]. For samples with a sufficiently high viral load (more than 1000 copies/mL), reverse transcription and PCR were performed, followed by Sanger sequencing of the nucleotide sequences of the *Pol* gene region encoding the protease (PR) and part of the reverse transcriptase (RT) using the AmpliSens HIVResist-Seq (Central Research Institute of Epidemiology, Moscow, Russia) commercial kit, including two steps of PCR followed by Sanger sequencing reaction. For HIV genotyping, we used a 1302 nucleotide sequence spanning the *Pol* gene (nt. 2253–3554). Coordinates given for the data represent the GenBank entry for HIV HXB2 (K03455.1). Analysis of sequence reaction products was performed using an ABI Prism 3500 genetic analyzer (Applied Biosystems).

Nucleotide sequences were aligned using the MEGA 11.0 program using the ClustalW algorithm [22]. To construct phylogenetic trees and subsequent phylogenetic analysis, the neighbor-joining algorithm and Tamura–Nei model were used, which made it possible to optimize trees in accordance with the balanced minimum evolution criterion. When assessing the reliability of phylogenetic relationships, we used multiple generations of samples using the bootstrap method for 1000 independent constructions of each phylogenetic tree. Genotyping of the studied strains was carried out in parallel using the REGA HIV-1 Subtyping Tool 3.0 program (https://hivdb.stanford.edu/page/hiv-subtyper/) (accessed on 3 September 2024) based on analysis of phylogenetic relationships with reference sequences from the GenBank international database [23].

Analysis of HIV-1 genetic sequences for the presence of drug resistance mutations was performed using the Stanford database, algorithm version 9.6 (https://hivdb.stanford.edu/hivdb/by-sequences/). Mutation profiles were analyzed by constructing line diagrams using Linear Diagram Generator software (https://www.cs.kent.ac.uk/people/staff/pjr/linear/index.html) [24].

Statistical data processing was carried out using the MS Excel 2016 and GraphPad Prizm 5.0 (GraphPad Software, Inc., San Diego, CA, USA) software packages. The “exact” Clopper–Pearson interval was used to estimate statistical uncertainty. Results are represented as a proportion indicating 95% confidence interval (95% CI). The Fisher exact test, or Yates-corrected chi-squared test, was used to evaluate the statistical significance of numeric data obtained during paired comparison, depending on sample characteristics. A probability value of *p* < 0.05 was taken as the statistical threshold of significance.

## 3. Results

**Figure 1:**
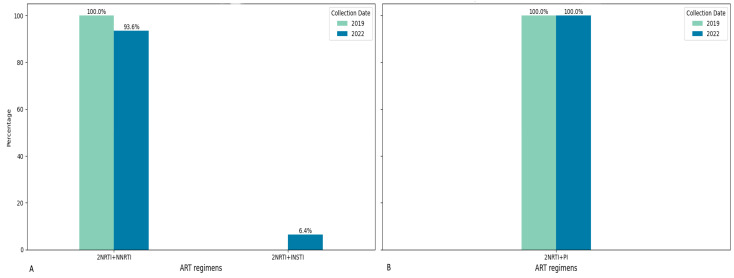
Ratio of examined patients with different first- and second-line ART regimens in different years of material collection. (**A**) First line of ART; (**B**) second line of ART.

**Figure 2:**
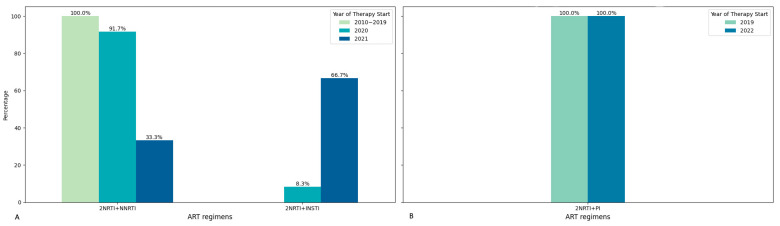
Ratio of patients with different first- and second-line ART regimens by year of treatment initiation. (**A**) First line of ART; (**B**) second line of ART.

Among patients seen in 2019 and 2022, the most common first-line ART regimen was a combination of two NRTIs and one INSTI. Among the patients whose material was obtained in 2022, some of them also received INSTI as part of the first-line regimen. Second-line ART was represented by one variant of the treatment regimen: 2NRTI + PI (it was only one combination of ARD: AZT + 3TC + LPV/r). The year of ART initiation varied in the patients studied from 2010 to 2021. At the same time, INSTIs were administered to patients only from 2020, and from 2010 to 2019, only one first-line ART regimen variant was administered in the studied patients: 2NRTI + NNRTI (Figure 1 and Figure 2).

**Figure 3:**
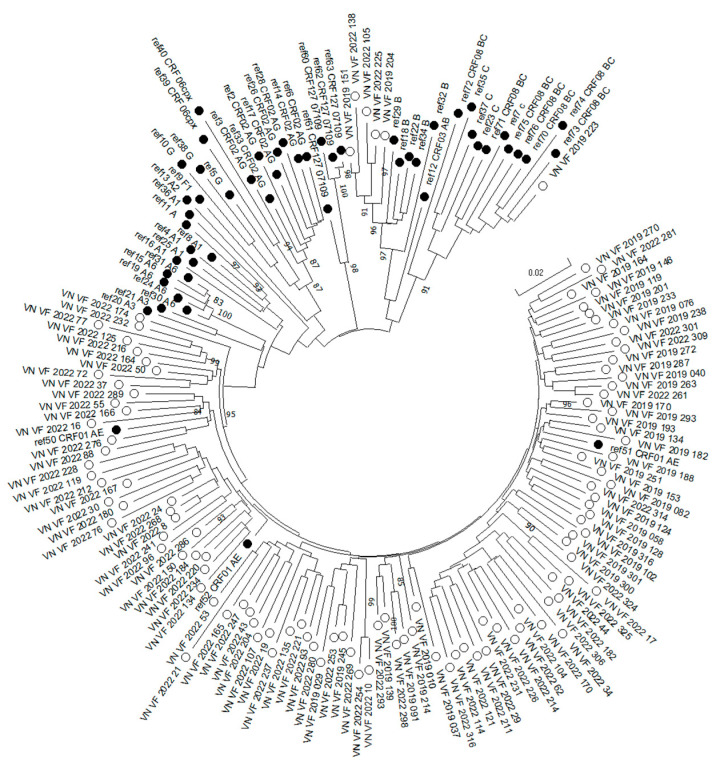
Phylogenetic analysis of viral nucleotide sequences (HIV *Pol* gene fragment) from HIV-infected persons in Vietnam relative to GenBank reference sequences. Reference sequences are designated as “ref” and indicate the sample genotype. Strains are marked as follows: black circles—reference sequences from GenBank; white circles—studied in this work sequences. Bootstrap values ≥ 70% are given.

The genetic sequences of the *Pol* gene of HIV isolates from 116 patients (38 from patients in 2019, 78 from patients in 2022) were successfully obtained. The nucleotide sequences of 116 HIV-1 samples were obtained and submitted to GenBank (numbers PP327226-PP327263, PQ126167-PQ126244). Subtype was determined for all samples. For this, data were obtained by genotyping using the REGA HIV Subtyping Tool 3.0 and the jumping profile Hidden Markov Model (jpHMM), and the results of a phylogenetic study (Figure 3) were used.

CRF01_AE was the most frequent genetic variant, occurring in 94.83% (95% CI: 89.98–98.08%) of the patients studied. In addition to this recombinant form, which is specific to the region, subtype B (4.31%, 95% CI: 1.41–9.77%) and CRF08_BC (0.86%, 95% CI: 0.02–4.71%) were found.

Among all patients, 49.14% experienced a change in their ART regimen. For patients with ART failure in 2019, three first-line ART regimens were used, with the most common being TDF + 3TC + EFV (64.71%). In 2022, four different first-line ART regimens were identified, one of which included DTG, though the most common regimen remained TDF + 3TC + EFV (75.00%). The second-line therapy involved three regimens in 2019 and five in 2022. The most common second-line ART regimen across both years was AZT + 3TC + LPV/r, used by 76.47% of patients in 2019 and 50.00% in 2022. In total, all patients analyzed in 2019 and 93.6% in 2022 were treated with (exposed to) NRTI + NNRTI-based regimens. Specifically, for NRTIs, AZT-based regimen usage rose from 50% in 2019 to 74.36% in 2022, while TDF-based regimen usage increased slightly from 50% to 56.4% over the same period. PI-based regimen usage was 44.74% in 2019 and increased to 51.28% in 2022. The average duration from ART initiation to virological failure was 5.45 years in 2019 and 4.06 years in 2022.

For patients who did not experience a change in treatment despite inefficacy, four different ART regimens were noted in 2019, with TDF + 3TC + EFV being the most prevalent (66.67%). In 2022, nine regimens were identified, with AZT + 3TC + NVP being the most common (26.32%). It is important to note that these regimens were not always prescribed in the same year that treatment inefficacy was detected; some patients remained on these first-line ART regimens for periods ranging from 1 to 8 years.

**Figure 4:**
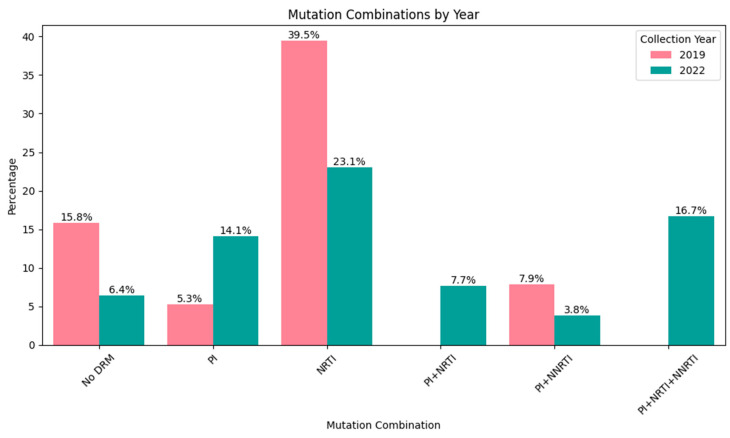
Frequency of HIV variants resistant to different classes of antiretroviral drugs and their combinations.

In 90.52% (95% CI: 83.67–95.17%) of cases, at least one HIVDR mutation was detected: 84.21% (95% CI: 68.75–93.98%) in 2019 and 93.59% (95% CI: 85.67–97.89%) in 2022. Among these, four cases exhibited only minor PI resistance mutations (3.81%, 95% CI: 1.05–9.47%). Drug resistance mutations across three classes of antiretroviral drugs were observed: protease inhibitors (PIs), nucleoside reverse transcriptase inhibitors (NRTIs), and non-nucleoside reverse transcriptase inhibitors (NNRTIs). The most common mutations involved resistance to both NRTIs and NNRTIs (32.38%, 95% CI: 23.57–42.21%) (Figure 4). In 2019, a significant proportion of isolates were resistant to only one class of drugs: NRTIs (18.75%, 95% CI: 7.21–36.44%) or NNRTIs (28.13%, 95% CI: 13.75–46.75%). By contrast, in 2022, the most common drug-resistant variants were those resistant solely to PIs (15.07%, 95% CI: 7.77–25.36%), and multidrug-resistant (MDR) isolates involving three drug classes were observed only in 2022 (17.81%, 95% CI: 9.84–28.53%).

From 2019 to 2022, there was a significant increase in the proportion of DR isolates with resistance to PIs, rising from 18.75% (95% CI: 7.21–36.44%) to 45.21% (95% CI: 33.52–57.3%) (φ* = 0.00403, *p* < 0.05).

**Figure 5:**
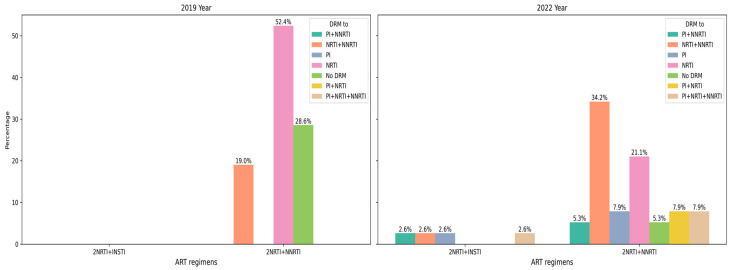
Occurrence of ARP resistance in patients with different ART regimens with one line of treatment.

**Figure 6:**
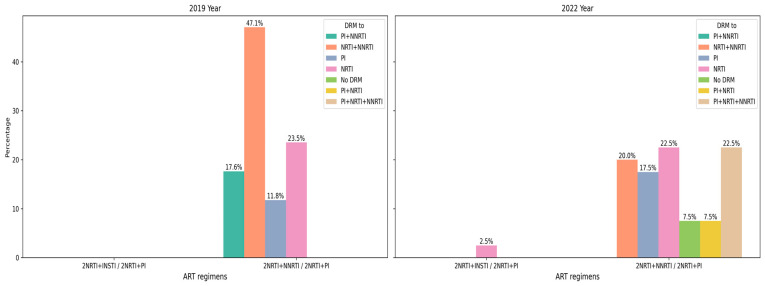
Occurrence of ARP resistance in patients with different ART regimens with two lines of treatment.

Virus strains obtained in 2019 from patients with a single line of ART demonstrate the absence of any supervisory drug resistance mutations in 28.6% of cases, with the remaining cases showing either isolated resistance to NRTIs or combined resistance to NRTIs and NNRTIs, which is broadly consistent with the treatment regimens that have a history of use in these patients. The same group in 2022 shows a much wider range of drug resistance variants, including ARPs that have no history of use (Figure 5 and Figure 6).

For patients with two treatment regimens, no such differences were found; in 2019, no strains without drug resistance mutations were detected in these patients, and only mutations corresponding to the prescribed drugs were detected.

**Figure 7:**
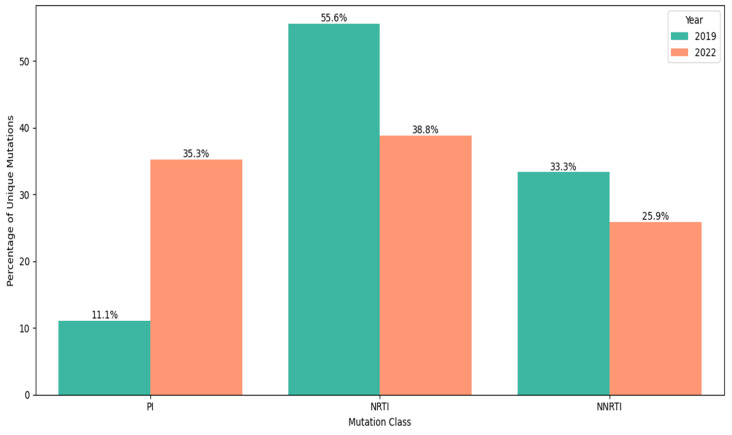
The proportion of resistance mutations for different drug classes among the total diversity of mutations identified.

In total, the number of patients with DR mutations among patients with ART failure in 2019 and 2022 differed slightly, but changes in the overall structure of drug resistance mutations are noteworthy (Figure 7). During the study period, a total of 92 different mutations associated with resistance to antiretroviral drugs (ARDs) were identified. NRTI DR mutations were the most diverse (39.13% of all mutations) but their proportion changed significantly from 2019 (55.56%) to 2022 (39.29%). These changes are obviously associated with a significant increase in the diversity of PI DR mutations, which accounted for 11.11% of all mutations in 2019 and 34.52% in 2022 (φ* = 0.01304, *p* < 0.05). These changes in the structure of mutations are also reflected in their prevalence. In 2022, there was a significant increase in the incidence of DR mutations for PI compared to 2019, both individually and in combination with resistance mutations for other drug classes.

**Figure 8:**
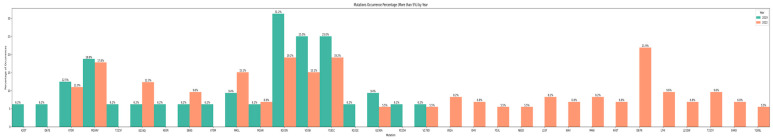
Occurrence of the most common drug resistance mutations for NRTIs and NNRTIs.

Some changes were also observed in the prevalence of individual mutations in 2022 compared to 2019. Since mutations associated with PI DR were practically not detected in 2019, it is not possible to conduct a comparative analysis of their prevalence in different years. In 2019, the only major mutation identified was M46I/L, found in 5.3% of HIV-infected individuals. In contrast, 2022 saw a range of major mutations associated with resistance to PIs, including V82A/S/T (10.26%), M46I/L and I54V/S/L (8.97%), G48M/V (6.41%), I84V (5.13%), I47V, and L90M (3.85%). Notably, 17.9% of patients in 2022 developed two to three major PI mutations, along with at least one to five accessory mutations. A comparative analysis of the prevalence of mutations of drug resistance to nucleoside and non-nucleoside reverse transcriptase inhibitors showed that the most noticeable changes occurred in the prevalence of some NRTI DR mutations (Figure 8).

These changes are primarily associated with an increase in the prevalence of AZT resistance mutations (TAM). In 2019, TAMs were not dominant in the structure of DR mutations. The most common at that time was the M184V mutation associated with resistance to 3TC. In 2022, the most common mutation was TAM D67N; its frequency of detection increased from 3.13% up to 21.92% (φ* = 0.01935, *p* < 0.05).

**Figure 9:**
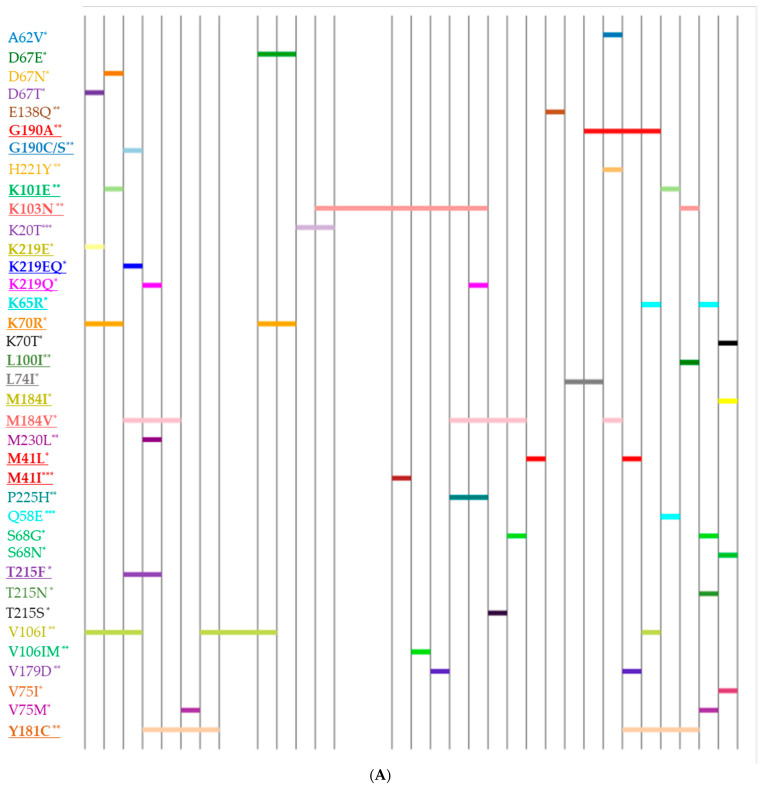

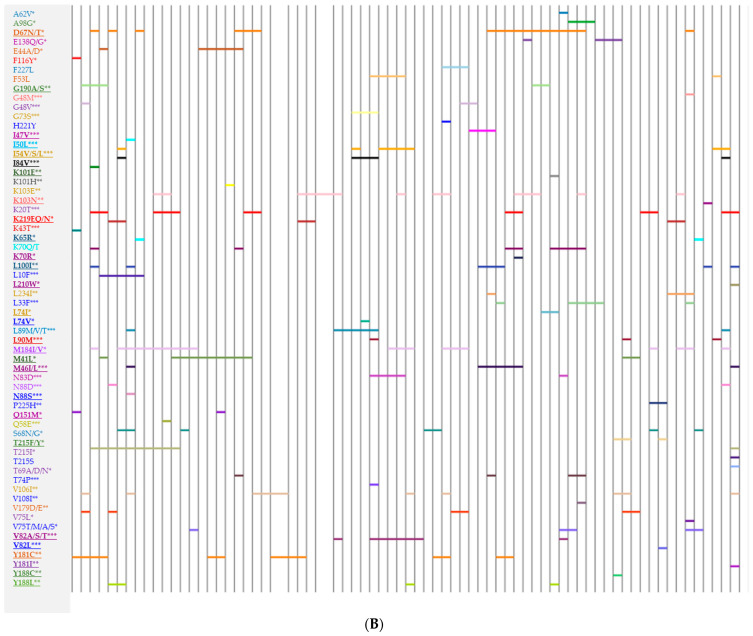
Line charts showing observed patterns of drug resistance mutations for NRTIs. Vertical bars represent DRM profiles. Horizontal marks represent a specific mutation in the profile. Bold underlines indicate major mutations. *: mutations to NRTIs, **: mutations to NNRTIs, ***: mutations to PIs. (**A**) 2019; (**B**) 2022.

An increase in the occurrence of TAM can also be noted when studying the profiles of drug resistance mutations and the mutation patterns detected in them (Figure 9). In 2022, viral isolates were identified containing mutation patterns along the TAM-1 pathway, including the T215Y mutation, which was absent in 2019. The most common TAM, D67N, occurred with various mutation patterns in the studied profiles, not specifically associated with any particular combination. Additionally, more than two mutations of the TAM-2 pathway and the accessory TAM (E44A/D), known for contributing to reduced NRTI susceptibility in combination with other TAMs, were exclusively detected in 2022. Additionally, the T215Y, L210W, and Q151M complex was observed only in 2022, with proportions of 8.97%, 6.41%, and 2.56%, respectively.

## 4. Discussion

The observed diversity in the treatment regimens prescribed to patients indicates a change in the practice of prescribing a combination of NTRI and INSTI as a first-line therapy regimen. From 2020 onwards, there has been an increase in the prescription of 2NRTI + INSTI regimens. At the same time, the second-line regimen remains unchanged in all years and includes one PI and two NRTIs.

Note the change in the spectrum of drug resistance mutations in patients in 2019 and 2022. These changes are particularly pronounced in patients with a single line of ART. It can be observed that in 2022, patients were resistant to more drug combinations, including those that were not recorded as being used in these patients. Certainly, the widening of the spectrum of drug resistance in 2022 can be explained by less careful adherence to treatment in patients and likely multiple interruptions in drug administration. However, this does not explain the presence of resistance mutations in patients to drugs that they were not taking. In this situation, we can assume an increased role of primary drug resistance or insufficient fixation of drug use in the patient’s history.

As in most parts of the world, the HIV epidemic in Vietnam began with injecting drug users and then spread to the general population, with heterosexual transmission predominating [25], although a relatively recent increase in HIV incidence among homosexual men has been recorded [26].

Regarding HIV-1 subtypes, our findings align with other studies in Vietnam, which indicate that CRF01_AE remains the predominant subtype. Additionally, minor subtypes such as B, C, or various genovariants of CRF01_AE have also been identified [16,21,27,28]. The highest prevalence of CRF01_AE has also been shown in Southeast Asian countries [29]. In Vietnam, a new circulating recombinant form of HIV-1 CRF127_07109 was identified in northern Vietnam in 2024 from samples collected from Dec 2019 to Jul 2022 [30]. In our study, while we employed four sequences of this subtype as references for building the phylogenetic tree, none of the samples we examined actually belonged to this particular subtype.

Since 2015, Vietnam has implemented a policy allowing all individuals diagnosed with HIV to start ART immediately, regardless of their CD4 count [12]. Previously, patients had to wait until their CD4 count met specific criteria, delaying treatment until the disease advanced. Our study revealed an average wait time of 2.22 years from HIV diagnosis to ART initiation, with some individuals waiting up to 7 years. This delay can significantly impact disease progression and patient outcomes [31,32]. In addition, among the study population, 68 HIV cases were detected from 2015 onwards, yet only 20 individuals (29.4%) initiated ART at the time of their diagnosis. In our study, the majority of patients initially received TDF + 3TC + EFV as their first-line ART regimen, consistent with national guidelines in place before November 2019 [12,13]. From December 2019 onwards, TDF + 3TC + DTG has been recommended as the preferred first-line ART regimen [14]. However, our findings indicate that since 2020, only three out of eighteen HIV-infected individuals who started ART received DTG-based regimens. These findings suggest that despite the policy change, a significant proportion of the study participants in the Mekong Delta region, who were experiencing treatment failure, did not initiate treatment promptly or with the recommended preferred regimens. A systematic review and meta-analysis pooling data from 29 studies across 15 countries, involving 34,937 participants, revealed that the prevalence of delayed ART initiation was 36.1% (95% CI: 29.7–42.5%). When stratified by gender, the review found that the male population had a slightly higher prevalence (delayed art) compared to the female population [33]. In our study, males also constituted the majority, comprising 69% of the participants.

This study investigated the occurrence of HIVDR among patients with suspicious clinical manifestations and/or those with insufficient adherence who experienced virological failure, defined as a single instance of high viral load (≥1000 copies/mL) detected at any time during their ART. In 2022, virological failure using this criterion was observed in 78 out of 326 individuals (23.9%, 95% CI: 19.4–28.9%), which was significantly higher than the 38 out of 316 patients (12.0%, 95% CI: 8.7–16.1%) in 2019 (OR = 2.3; 95% CI: 1.47–3.61; *p* = 0.0001). Additionally, the average time from ART initiation to virological failure was shorter among patients in 2022 (4.06 years) compared to those in 2019 (5.45 years).

In recent years, various factors, including the impact of the COVID-19 pandemic, have caused delays in the supply of ARV drugs through health insurance, as well as reduced and untimely HIV viral load testing. Key challenges include the effects of COVID-19 prevention policies, patient unemployment leading to interruptions in health insurance coverage, and disruptions in the continuity of ARV treatment and necessary tests funded by health insurance. Additionally, difficulties in the procurement and bidding processes for health insurance drugs have arisen due to the COVID-19 pandemic and amendments to the Law on Bidding of Vietnam, as well as those funded by aid or the state budget, which have led to delays and complications in the supply of certain medications. Although this has complicated drug coordination, there has been no reported shortage of drugs for patients.

For HIV viral load testing, the scarcity or slow supply of test kits has resulted in a lower number of individuals on ARV treatment undergoing these crucial tests [34,35,36]. The proportion of patients with unsuppressed viral loads in our study was higher than in previous national surveys conducted in 2014 and 2017–2018, which reported values of less than 5% among patients on ART for 12 months and over 48 months [16] and 5.4% among those receiving ART for 36 months or longer [17]. In those national surveys, participants were selected based on their routine follow-up schedule for viral load testing, typically every 12 months, leading to a higher proportion of viral suppression.

In contrast, our study focused on patients with virological failure, suspicious clinical manifestations, and/or insufficient adherence. This difference may explain the higher proportion of viral non-suppression found in our study. A similar study conducted between 2006 and 2009 reported up to 36.96% of patients with immunological and/or clinical failure presenting with viral non-suppression [37], a figure notably higher than those in our current study. During the 2006–2009 period, HIV-infected individuals were only eligible to start ART once they met specific immunological or clinical criteria, such as a significant drop in CD4 count or the onset of opportunistic infections. As a result, many patients initiated treatment at more advanced stages of HIV infection, which likely contributed to higher rates of virological failure and unsuppressed viral loads. Moreover, the available ART regimens at that time may have been less effective or less accessible than those in current use, further contributing to the higher rates of treatment failure. Indirectly supporting this assumption is the fact that in a study on antiretroviral drug resistance mutations among patients failing first-line treatment in Hanoi, HIV drug resistance mutations were detected in 90.7% of subjects whose samples were obtained between 2006 and 2016 [38]. Despite the impact of COVID-19, our results still reflect the efficacy of advancements in national treatment protocols, earlier ART initiation, and better patient management over time.

Among individuals experiencing suspected virological failure, we observed 11 patients (9.48%) whose samples did not feature any resistance-conferring mutations, suggesting that other factors may have contributed to therapeutic failure. All of these patients had exclusively used a single, first-line regimen up to the point of detecting viral non-suppression. This situation could include issues such as poor adherence to medication, drug interactions, inadequate drug levels in the body, or even underlying health conditions affecting treatment efficacy. In contrast, the prevalence of drug resistance mutations was 4.6% among PLHIV not previously receiving ART examined in 2019–2022, decreasing from 6% in 2019/2020 to 1.3% in 2022 [39].

Our finding was lower than the data from the Vietnam national surveillance of acquired drug resistance (ADR) in 2017–2018, which revealed 21.4% among patients on ART after 12 months and 11.1% among those on ART for over 48 months [16] but higher than those in 2014, which showed that 5% of individuals on ART for more than 36 months did not present any mutation [17]. However, HIVDR testing is unavailable throughout the Mekong Delta region in Vietnam. This situation raises concerns about the potential for unnecessary switches to second-line ART for patients whose treatment failure is determined solely based on virological criteria. Since 2021, this issue has been further compounded by the COVID-19 pandemic, which led to social distancing measures and disruptions in healthcare services, as well as other challenges that have interrupted the routine delivery of HIVDR testing during clinical follow-ups in Vietnam to date.

Our study demonstrated that a single “high viral load” finding at any point during ART is associated with resistance, with a significant increase observed in both the detection of HIVDR mutations and changes in mutation profiles, between 2019 and 2022. Since all patients were exposed to NRTI and NNRTI drugs, resistance was most commonly observed against these two classes concurrently, at 37.5% and 30.13% in 2019 and 2022, respectively. Our findings were lower than those reported in previous national surveillance studies in Vietnam, where resistance rates reached 50% after 12 months, 80.2% after more than 48 months in 2017–2018 [16], and 79.9% among individuals on ART for 36 months or longer in 2014 [17]. The data revealed a decrease in the percentage of patients presenting resistance to NRTIs and NNRTIs from 2019 to 2022. Notably, the decrease in NNRTI resistance was statistically significant (OR = 0.32, χ^2^ = 5.43, *p* < 0.05). Additionally, there was a significant increase in the proportion of DR isolates resistant to PIs, rising from 18.52% in 2019 to 45.21% in 2022 (φ* = 0.00403, *p* < 0.05). These changes may be attributed to the rise in the rate of patients treated with PI-based regimens during this period.

Notably, among patients experiencing failure in 2022, we also identified 17.81% with resistance to triple drug classes, a phenomenon not observed among patients in 2019 or in our previous national surveillance [16,17]. The emergence of mutations resistant to PIs while still retaining mutations resistant to NNRTIs presents a complex challenge in HIV/AIDS treatment and quality of life, as the virus may not respond adequately to standard first-line or second-line therapies. Some studies have indicated that one reason for the high level of PI resistance is the lack of viral load monitoring, which results in the late detection of virological failure and the accumulation of PI resistance mutations [40,41,42]. 

Managing dual-resistant HIV requires a multi-faceted approach combining clinical care and patient education. However, in Vietnam, limited use of these third-line options complicates this challenge. Furthermore, the pandemic has severely limited access to crucial viral load tests, leaving some patients stuck with ineffective treatments that could ultimately lead to treatment failure.

As expected, M184I/V was the most common NRTI mutation observed in our study, consistent with findings from other studies [7,18,38]. Our findings indicate that the prevalence of TAMs increased in 2022, particularly with a significant rise in the frequency of the D67N mutation, which increased from 3.13% to 21.92%. Other mutations, including M41L, K219Q, T215Y, and L210W, were also more prevalent among patients in 2022. This rise may correlate with the increased use of AZT-based regimens, which grew from 50% in 2019 to 74.36% in 2022. García-Lerma et al. demonstrated that the D67N and K219Q/E mutations rapidly evolve to confer ZDV resistance in vitro and exhibit high replicative fitness in the presence of ZDV [43].

The accumulation of multiple mutations in both TAM pathways and the Q151M complex in 2022 suggests prolonged virological failure. Studies have demonstrated that accumulated multiple TAMs and the Q151M complex are associated with reduced phenotypic drug susceptibility [44,45,46]. Despite the near-universal development of M184V during virological failure, which reduces the impact of TAMs on AZT and TDF susceptibility [47], our study found that 21.1% of patients had TAMs without concurrent M184V mutation. Although over half of patients were treated with (exposed to) TDF, the K65R mutation (which is usually selected under TDF pressure) was detected in only two cases in 2019 and one case in 2022. Viktor von Wyl et al. provided evidence for a protective effect of TAMs, particularly the TAM-2 pathway, against the emergence of K65R [48]. This may help explain the very low proportion of K65R observed in our study.

Although there was no significant difference in the proportion of patients receiving PI-based regimens between these two years (44.74% in 2019 to 51.28% in 2022; OR = 0.76, χ^2^ = 0.44, *p* = 0.51), our data indicate notable changes in the accumulation of mutations. Specifically, there was a significant increase in the diversity of PI drug resistance mutations, which accounted for 11.11% of all mutations in 2019 and rose to 34.52% in 2022.

Overall, the increased resistance seen in patients with virological failure in 2022, compared to 2019, highlights the potential impacts of the COVID-19 pandemic. Disruptions in healthcare services have affected the prevention and treatment of various diseases, including HIV. Challenges such as limited access to laboratory monitoring, clinical visits, and ART may have led to suboptimal care for HIV infection and other conditions among people living with HIV [34,35,49]. In people living with HIV, inconsistent adherence to ART stands out as the primary predictor of virological failure, drug resistance, disease progression, and mortality.

It is clear from the above that the COVID-19 pandemic had an impact on the prevalence of drug-resistant HIV variants. This was a consequence of diverting major health system capacity to the pandemic. There are reports that previous epidemics, such as the SARS epidemic, also had a significant impact on the epidemiology of HIV infection [50]. Furthermore, it is evident that it is not only epidemics that can have such an impact on the effectiveness of HIV control. Also, military conflicts and associated global migration can play an important role in epidemic processes [51,52]. Thus, any situation associated with humanitarian disasters leads to an extremely high burden on the healthcare system, which affects the provision of care to patients, including patients with HIV infection, and there are interruptions in treatment that inevitably lead to drug resistance of the virus.

Our study had certain limitations. As it was a cross-sectional study rather than a cohort, the samples collected in 2019 and 2022 were from different patients. However, the HIV treatment program has been consistently implemented nationwide, including in the Mekong Delta region. The key difference between these two years was the impact of the COVID-19 pandemic in Vietnam from 2020 to 2022, which posed significant challenges to the healthcare system. Other limitations include the absence of comparative data on treatment initiation between patients with and without virological failure, the lack of detailed information regarding immunological and clinical status, as well as the adherence of the patients enrolled in the study, particularly the specific obstacles they faced during treatment at that time. In addition, although there were five patients who received integrase inhibitors, we did not analyze for HIVDR by the integrase gene. Despite these limitations, our study provides important data on the drug resistance situation among patients receiving ART, a topic that has seen limited publication in Vietnam since the onset of COVID-19.

## 5. Conclusions

The COVID-19 pandemic has significantly affected the livelihoods of people living with HIV from vulnerable populations, with consequences in various aspects of their lives, including access to and adherence to antiretroviral therapy (ART). Among patients in the Mekong Delta region, especially after the onset of the pandemic, there is a high incidence of lack of viral suppression, as evidenced by the increasing proportion of patients with high viral load on ART. Also, HIV drug resistance is more common in patients seen after the pandemic, and the spectrum of mutations associated with resistance is expanded. Taken together, these findings confirm the significant impact of pandemics such as this one on the effectiveness of HIV control. Our study highlights the current challenges that Vietnam’s HIV/AIDS treatment program must address in the post-pandemic period in order to sustain the gains made and achieve the goals of the country’s HIV prevention strategies.

## Data Availability

Data are available on request from the authors.
